# Validation of the Mayo alliance prognostic system for mastocytosis

**DOI:** 10.1038/s41408-019-0179-7

**Published:** 2019-02-11

**Authors:** Francesco Mannelli, Francesca Gesullo, Giada Rotunno, Annalisa Pacilli, Lisa Pieri, Paola Guglielmelli, Alessandro M. Vannucchi

**Affiliations:** CRIMM, Centro di Ricerca e Innovazione per le Malattie Mieloproliferative, Azienda Ospedaliera Universitaria Careggi, Dipartimento di Medicina Sperimentale e Clinica, Università degli Studi, Firenze, Italy

To the Editor:

Systemic mastocytosis (SM) is a myeloproliferative disorder characterized by extreme heterogeneity of clinical manifestations, course and prognosis. According to the WHO 2016 classification, selected clinical findings are useful for distinguishing aggressive from indolent variant, a distinction that drives therapeutic decisions^1^. Additional clinical and laboratory variables were then shown to correlate with survival, including advanced age, elevated beta-2-microglobulin and alkaline phosphatase (ALP) levels^2,3^. Also, mutations of selected genes, usually associated with “high risk” features in other myeloid neoplasms^4,5^, were identified, that provide clinically relevant information about disease’ course; in particular, mutation in *SRSF2*, *ASXL1*, *RUNX1*, and *CBL* were shown to be prognostically relevant^6–8^, and were also validated recently in our patients’ cohort^9^. Conversely, unlike other myeloproliferative neoplasm, particularly myelofibrosis^10^, cytogenetic abnormalities in patients with SM are infrequent and have poor prognostic relevance^11^. By integrating the above prognostically relevant clinical and molecular parameters in a large cohort of SM patients seen at Mayo Clinic (*n* = 580), Pardanani et al. recently derived two prognostic models that may find wide clinical application: the first one was based upon clinical parameters only, the second enlisted both clinical and genomic findings^9^. Variables enlisted in the clinical model include SM variant (advanced versus indolent/smouldering SM), age > 60 years, platelets < 150 × 10^9^/L, sex-adjusted anemia and serum alkaline phosphatase above normal range, that are complemented by adverse mutations (*ASXL1, RUNX1, NRAS*) in the hybrid clinical-molecular score.

The aim of the current study was to assess the performance of the Mayo Alliance Prognostic System (MAPS) models in a real-life setting at our Center. After approval from the institutional review board, we interrogated our database for the availability of MAPS-related parameters, and finally applied the clinical-only model to 94 SM patients and the hybrid clinical-molecular model to 65 patients, out of a total of 127 patients with diagnosis of SM observed in the period from 2000 to 2018; information was last updated in August 2018.

Clinical and laboratory features of the study population are summarized in Table 1. According to WHO classification^1^, 82 of 94 (87.2%) patients were diagnosed with non-advanced forms, 78 (83.0%) were indolent and 4 (4.3%) smouldering SM. The 12 advanced cases included 9 aggressive variants (ASM, 9.6%), 2 with an associated hematological neoplasm (SM-AHN, 2.1%), and 1 mast cell leukemia (MCL, 1.1%). As expected from other reports in larger cohorts, compared with advanced SM, the patients with indolent/smouldering SM were younger, had higher hemoglobin and platelet count, lower leukocyte count, and lower levels of serum alkaline phosphatase and tryptase. Overall, 98% of the patients were found positive for the *KIT*D816V mutation. Additional myeloid mutations (*ASXL1, RUNX1*, and *SRSF2*) were assessed in 65 patients; for 21 of them, genotyping of *NRAS* was also available. Overall, we observed 6 cases with high risk mutations, all of which were included among advanced forms. The rate of adverse mutations (9.2%) was lower that reported by Pardanani and colleagues (21%), and is likely to be accounted for by a greater representation of advanced forms in their database. Fifty-eight percent of patients with advanced SM died, after a median follow-up of 23 months, as compared to 1.2% of indolent/smouldering SM, after a median follow-up of 50 months (*P* < 0.001).

**Table 1:** Clinical and laboratory features of 94 patients with systemic mastocytosis (SM) included in the study

Variables	Overall *n* = 94	Indolent/smouldering SM *N* = 82	Advanced SM *N* = 12	*p* value
Median age (range)	47 (17–80)	45 (17–80)	64 (23–78)	**0.01**
Age > 60 years; *n* (%)	26 (27.7)	19 (23.2)	7 (58.3)	**0.017**
Hemoglobin, g/dl, median (range)	13.7 (4.8–16.2)	14.2 (10.1–19.4)	11.1 (5.1–17.4)	**<0.001**
Anemia sex adjusted; *n* (%)	22 (23.4)	11 (13.4)	11 (91.7)	**<0.001**
Leukocyte count x 10^9^/l, median (range)	7.3 (2.0–39.8)	7.1 (3.2–17.1)	12.1 (2.0–39.8)	**0.022**
Platelet count x 10^9^/l, median (range)	232 (70–421)	263 (98–456)	79 (10–368)	**<0.001**
Platelet count < 150 × 10^9^/l; *n* (%)	14 (14.8)	3 (3.7)	10 (83.3)	**<0.001**
Serum tryptase ng/ml; median (range)	33.0 (3.1–7180)	29.0 (3.0–591)	162.5 (30.0–7180)	**<0.01**
Serum ALP, U/l; median (range)	75 (20–184)	90 (31–280)	148.5 (20–438)	**0.004**
Serum ALP > UNL; *n* (%)	21 (22.3)	14 (17.1)	7 (58.3)	**<0.001**
*KITD816V; n* (%) *N* *=* *88*	86 (97.7)	75 (97.4) *N* Evaluable = 77	11 (100) *N* Evaluable = 11	1.0
*ASXL1* mutated; *n* (%) *N* *=* *65*	3 (4.6)	0 (0) *N* Evaluable = 55	3 (30) *N* Evaluable = 10	**0.003**
*RUNX1* mutated; *n* (%) *N* *=* *65*	1 (1.5)	0 (0) *N* Evaluable = 55	1 (10) *N* Evaluable = 10	0.154
*NRAS* mutated; *n* (%) *N* *=* *21*	1	0 (0) *N* Evaluable = 16	1 (25) *N* Evaluable = 5	0.238
*SRSF2* mutated; *n* (%) *N* *=* *65*	1 (1.5)	0 (0) *N* Evaluable = 55	1 (10) *N* Evaluable = 10	0.154
Adverse mutations; *n* (%) *N* *=* *65*	6 (9.2)	0 (0) *N* Evaluable = 55	6 (50) *N* Evaluable = 10	**<0.001**
Median follow-up in months (range)	46 (2.1–209)	50.1 (2.1–209)	23.4 (2.1–115)	**0.022**
Deaths; *n* (%)	8 (8.5)	1 (1.2)	7 (58.3)	**<0.001**

*SM* systemic mastocytosis, *SM-AHN* systemic mastocytosis with an associated hematological neoplasm, *ALP* alkaline phosphatase, *UNL* upper normal limit, *N* means the number of patients for which the particular information was available

*p* values lower than significance threshold are highlighted by bold

**Fig. 1:**
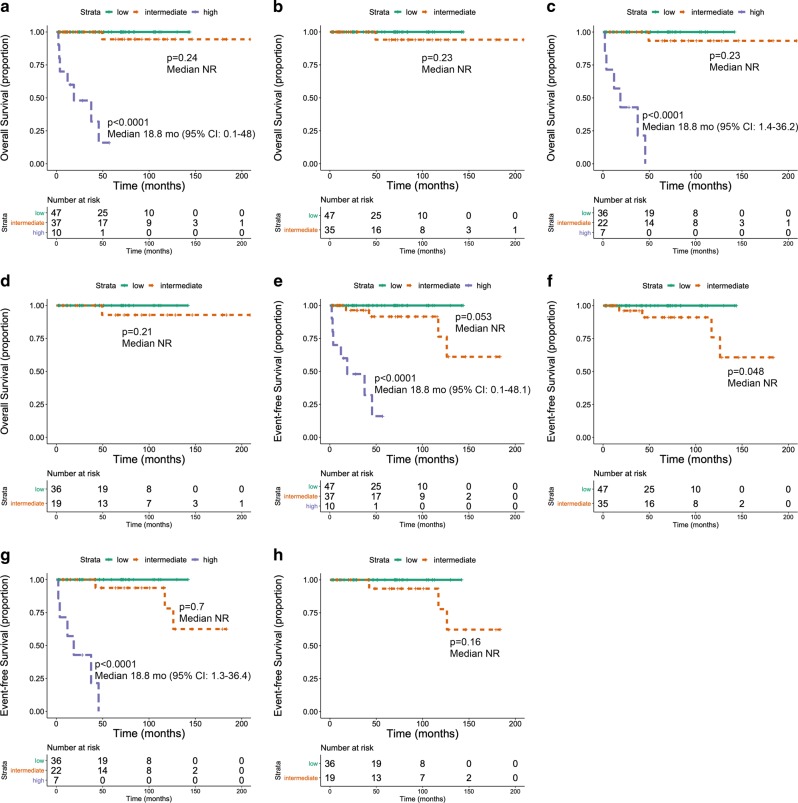
The panels from (**a**–**d**) illustrate the performance of the Mayo clinical-only score (**a**, **b**) and the hybrid clinical-molecular score (**c**, **d**) concerning overall survival for: the low, intermediate and high risk category (**a**, **c**), and the low and intermediate-risk category only (**b**, **d**). The panels from (**e**–**h**) illustrate the impact of the Mayo scores regarding event-free survival in the different risk categories, as above

Owing to the prevalence of indolent/smouldering cases in our cohort, we carried out a 3-tier stratification of patients according to the MAPS clinical and hybrid clinical-molecular model, by gathering patients with 1–2 and 3 to 5 risk factors in 2 categories only (intermediate and high risk, respectively) while patients with no risk factors were included in a low-risk category. Results of the analysis in our cohort excellently validated the performance of the MAPS score, overall resulting in a dramatic separation of patients with dismal overall survival (OS) and event-free survival (EFS) from others with very favorable course (Fig. 1). In particular, both models were able to identify an adverse risk category, with a median OS of only 18.8 months (95% CI, 0.1–48), significantly shorter than the intermediate and low-risk categories (OS not reached in both) (Fig. 1a, c for the clinical-only and hybrid model, respectively); the lack of a significant difference between low- and intermediate-risk patients can reasonably be ascribed to the limited number of events in our cohort mainly including not aggressive forms of SM, as exemplified by separate analysis of indolent/smouldering cases (Fig. 1b, d for the 2 models)

We also evaluated performance of the Mayo models using event-free survival (EFS) as an endpoint; events were death and progression from indolent to aggressive variant, that constitutes a major clinical event influencing long term outcome in SM (Fig. 1e–h). As shown in Fig. 1, the clinical-only model allowed prediction of shorter EFS in the entire cohort (*P* = 0.053; Fig. 1e) and, notably, in the indolent/smouldering cases (Fig. 1f; *P* = 0.048). The performance of the hybrid model at this regard was suboptimal, as it might be anticipated by the combined effects of limited number of cases and events (Fig. 1g, h).

In conclusion, this analysis allowed to confirm the general validity of risk stratification of patients with systemic mastocytosis according to the newly developed Mayo models; furthermore, we revealed its performance in predicting prognosis of intermediate category’ patients, especially using the clinical model. The proportionality between risk factors and outcome reaffirms the proof-of-principle underneath Mayo model.

The easy and prompt attainability of parameters included in the clinical model makes this particularly suitable for routine patients’ management. The use of the hybrid clinical-molecular model might be currently reserved to younger patients, where stem cell transplantation is an option, and should be definitely incorporated in clinical trials; also, it will be important to prospectively validate its performance in the era of new targeted agents like midostaurin^12^. It is also conceivable that further insights into the mutation landscape of SM patients might lead to improvement of the hybrid clinical-molecular score, and making it more widely available and enhancing its performance.
